# Use of fake identification to purchase alcohol amongst 15-16 year olds: a cross-sectional survey examining alcohol access, consumption and harm

**DOI:** 10.1186/1747-597X-5-12

**Published:** 2010-06-22

**Authors:** Michela Morleo, Penny A Cook, Mark A Bellis, Linda Smallthwaite

**Affiliations:** 1 Centre for Public Health, Liverpool John Moores University, Fifth Floor, Kingsway House, Hatton Garden, Liverpool, L3 2AJ, UK; 2 Warrington & Halton Trading Standards, Warrington Borough Council, Public Protection Services, Business Support Centre, New Town House, Buttermarket Street, Warrington, WA1 2NH, UK

## Abstract

**Background:**

Despite legislation and enforcement activities to prevent underage access to alcohol, underage individuals continue to be able to access alcohol and to do so at levels which put them at significant risk of alcohol-related harm.

**Methods:**

An opportunistic survey of 15-16 year olds (n = 9,833) across North West England was used to examine alcohol consumption, methods of access and related harms experienced (such as regretted sex). Associations between these were analysed using chi square and logistic regression techniques.

**Results:**

Over a quarter (28.3%) of 15-16 year old participants who drank reported having bought their own alcohol. One seventh (14.9%) of these owned at least one form of fake identification for which by far the most common purchase method was online. Logistic regression analyses showed that those who owned fake identification were significantly more likely to be male (AOR = 2.0; 95% CI = 1.7-2.5; P < 0.001) and to receive a higher personal weekly income (comparing those who received > £30 with those who received ≤ £10: AOR = 3.7; 95% CI = 2.9-4.9; P < 0.001). After taking into account differences in demographic characteristics and personal weekly income, ownership of fake identification was significantly associated with binge drinking (AOR = 3.5, 95% CI = 2.8-4.3; P < 0.001), frequent drinking (AOR = 3.0, 95% CI = 2.5-3.7; P < 0.001) and public drinking (AOR = 3.3, 95% CI = 2.5-4.1; P < 0.001) compared with those who did not own fake identification. Further, those who reported owning fake identification were significantly more likely to report experiencing a variety of alcohol-related harms such as regretted sex after drinking (chi square, all P < 0.001).

**Conclusions:**

Young people (aged 15-16 years) who have access to fake identification are at a particularly high risk of reporting hazardous alcohol consumption patterns and related harm. Owning fake identification should be considered a risk factor for involvement in risky drinking behaviours. Information on these hazards should be made available to schools and professionals in health, social and judicial services, along with advice on how to best to work with those involved.

## Background

The prevention of alcohol misuse and related harm has become an increasing worldwide concern[1], with alcohol being responsible for 4.0% of the global burden of disease[2], and 195,000 deaths in Europe annually[3]. Addressing consumption amongst young people is a particular priority [4,5], since their excessive consumption is associated with a number of acute risks including hospital admission [6], poor school performance [7], regretted sex [8] and offending [8-10]. Long-term risks are also apparent as those who have abused alcohol by 15 years are four times more likely to experience alcoholism in their lifetime [11]. In England between 1990 and 2006, self-reported quantities of alcohol consumed by 11 to 15 year olds who had drunk alcohol in the last week more than doubled from 42.4 to 91.2 grams [12]. The most recent figures cannot be directly compared with those from 1990-2006 because of changes in the way consumption has been calculated from 2007 onwards. However, the two most recent years' data (2007 and 2008) show another increase in quantities consumed by 11 to 15 year old drinkers from 101.6 to 116.8 grams [13].

As a result of the harms associated with drinking during childhood and adolescence, legislation has been established internationally in order to prevent access to alcohol by those who are deemed underage [14,15]. Thus combating harms associated with alcohol amongst young people is a public health priority in Europe [16]. In the UK, individuals under the age of 18 years cannot legally purchase alcohol. In addition, the reduction or prevention of underage alcohol consumption is a UK Government priority, referred to as a key aim both in the national strategy [17] and in alcohol licensing legislation [18]. Here, compliance with legislation can be verified through, for example, test purchasing exercises (where underage volunteers attempt to buy alcohol) [19-21]. Failure to comply with minimum age legislation can result in warnings, penalty notices for disorder, cautions, prosecution, licence and/or, where necessary, licence removal. Whilst data are available on the number of test purchasing operations and the number of failing venues [20], there is little intelligence on the effectiveness of such exercises in preventing underage purchase, the percentage of young people able to carry out a self-purchase, or how such self-purchases are conducted.

Literature from the United States shows that fake identification documentation is used by underage drinkers to access alcohol, and up to half of college students (aged under 21 years) may purchase alcohol in this way [22,23]. However, the US minimum purchasing age is 21 years, and so such studies may not reflect the situation in countries with a lower minimum purchase age (such as 18 years in Australia, Hungary and the UK, and 16 in Italy [14,15]). This paper explores the prevalence of self-purchase, of being asked for identification when attempting a self-purchase, and ownership of fake identification documents in the UK. It goes on to explore the source of their fake identification, the extent to which those who own fake identification are involved in risky alcohol behaviour, and the characteristics of those who own fake identification. In this way, we highlight the importance of tackling fake identification in reducing alcohol-related harm amongst 15-16 year olds. Further we discuss how this group can be identified more easily in order to develop appropriate interventions.

## Methods

A cross-sectional opportunistic (non-random) survey on alcohol consumption and access to alcohol was conducted in 2007 in schools amongst 14 to 17 year olds [8] by Trading Standards North West (a Government body who uphold trading regulations) in the North West of England, a region with significantly high levels of alcohol misuse compared with elsewhere in England [24]. The survey used closed self-completion questions covering a number of topics. Firstly, in order to understand the characteristics and experiences of those involved in risky behaviours, questions included: demographics; personal weekly income of the young person (for example, from pocket money and money received through employment); drinking frequencies; and quantities of alcohol consumed in a typical week. Details of drinking frequencies and quantities were used to inform the following categories: binge drinking (drinking five or more alcoholic drinks on one occasion at least once a week, a definition used to describe binge drinking in 15-16 year olds in large-scale European survey[25,26]) and frequent drinking (drinking at least twice a week). Whether the participant drank in public places was also included (drinking outside in streets, around shops and in parks) as a measure of the potential for social nuisance. To understand experiences of purchasing and access, participants were asked details of their sources of alcohol; whether they had been asked for identification when attempting to buy alcohol; and if they had used fake identification. Finally, participants were also asked questions regarding their experiences of alcohol-related harm in order to understand whether those who owned fake identification were more at risk of experiencing such incidents. Harms listed on the questionnaire (one question for each harm) related to: entering a car with a drunk driver; violence when drunk; regretted sex and memory lapses (episodes where the individual did not remember past events after drinking). The first three harms were binary questions, where the participant could select yes or no. Data on memory lapses were collected via a four point ordinal Likert scale (agree strongly, agree, disagree, disagree strongly) asking whether individuals felt that they tended to forget things after drinking, which was then categorised into those who agreed that they tended to forget about drinking and those who did not.

The survey was anonymous and was made available for schools in the North West to participate voluntarily through local Trading Standards services. No incentive was offered for participation. Sampling was intended to encompass a wide range of community types. Participating schools allowed pupils to voluntarily complete the questionnaires during normal school lessons. All aspects of the methodology complied with the Declaration of Helsinki and consent was provided through the regional trading standards board and participating schools[8]. In total, 140 schools in 19 unitary and upper tier local authorities took part in the survey (out of 22 such authorities in the North West region[27]), returning 11,724 questionnaires. Compliance levels were not recorded because the sample was intended to be opportunistic, with analysis focusing on relationships between variables that were recorded by individual participants[8]. Analyses were limited to 15-16 year olds, the largest age group surveyed, providing an analysed sample of 9,833.

Data were entered by Ci Research into SPSS v14, and then cleaned and analysed by Liverpool John Moores University (SPSS v17). Deprivation was allocated according to the Index of Multiple Deprivation (IMD)[24,28] for their resident Lower Super Output Area (geographical areas with an average population size of approximately 1,500 individuals)[8]. (IMD is a national measure and is calculated through the use of factors such as income, employment, skills and training, and barriers to housing. Allocated scores are then assigned to super output areas[28]). We assigned IMD scores through either their full (n = 4,158) or partial postcode (n = 1,744) where provided. For those without a postcode (n = 2,063), the postcode of their school was used as a proxy, a method employed successfully elsewhere [29]. (Here, a strong correlation was identified between deprivation scores derived from our sample's postcodes with those derived from that of the school; P < 0.001[8]). Individuals who provided insufficient data (n = 298) were excluded from geographic analyses. The scores were then categorised into IMD quintiles. Participants' income was calculated through the use of three questions asking for details of amounts of money obtained from parents, work and other sources. We totalled the sums provided.

Analysis incorporated chi square and logistic regression techniques. Logistic regression was used firstly to estimate the likelihood of ownership of fake identification (from sex, age, deprivation and personal weekly income), and secondly to assess the importance of owning fake identification in relation to experiences of harmful consumption patterns (controlling for demographic characteristics).

## Results

The majority of pupils (84.0%) drank alcohol at least occasionally. Of the drinking participants, over a third (36.3%) reported binge drinking; over a quarter (28.7%) reported frequent drinking; and 55.2% reported drinking in public places. The most common method of accessing alcohol by drinkers was through friends and family aged over 18 (50.3%) and parents (49.4%), followed by self-purchase (28.3%). Female drinkers were significantly more likely to access alcohol via friends or family (aged both over and under 18 years) and from parents compared with males (chi square = 108.3, 10.1, 17.4 respectively, all P < 0.001; all df = 1), who in turn were more likely to buy alcohol themselves (chi-square = 4.7; P = 0.029; df = 1).

When drinking participants who reported self-purchase were asked whether alcohol outlets had ever requested identification at the point of purchase, over half (56.4%) said that this had occurred at least once. Male drinkers who self-purchased were significantly more likely to report that they had ever been asked for identification (61.5%) than females (51.0%; chi-square = 53.2, P < 0.001; df = 1). One seventh (14.9%; 342) of those who drank and self-purchased owned fake identification (equivalent to 3.5% of total sample). In fact, self-purchasing drinkers who reported ever being asked for identification were significantly more likely to own at least one form of fake identification (19.0% compared with 9.5% of those who had not been asked; chi-square = 39.8, P < 0.001; df = 1). Of those who owned fake identification (520; 5.3% of the overall sample), two thirds reported that they had self-purchased alcohol.

**Figure 1:**
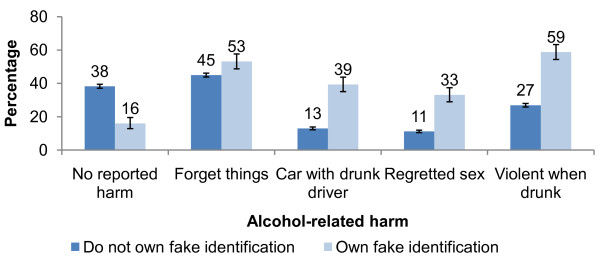
Percentage of alcohol-consuming 15 and 16 year olds experiencing alcohol-related harm* by fake identification ownership**. Error bars on the figure represent 95% confidence intervals. * Participants were asked whether they had ever experienced one (or more) of the listed harms after drinking alcohol. Those who did not report experiencing any harms are listed here as no reported harm (n = 7,442). Harms explored include: forgetting things after drinking (n = 7,743), entering a car with a drunk driver (n = 7,822), having regretted sex after drinking (n = 7,286) and being violent when drunk (n = 7,673). **All of the comparisons between those who do and do not have fake identification are significant (chi square analysis, all P < 0.001; df = 1).

Logistic regression was used to account for confounding factors among alcohol consumers. Those with fake identification were more likely to be male (AOR = 2.0; 95% CI = 1.7-2.5; P < 0.001; Wald chi-square = 48.0; df = 1) than female and those receiving a personal weekly income of more than £30 were more likely to own fake identification than those with an income of £10 or less (AOR = 3.7; 95% CI = 2.9-4.9; P < 0.001; overall Wald chi-square = 117.6; df = 3; Table 1). There was no clear relationship with deprivation and no significant difference between 15 and 16 year olds. By far the most common method of obtaining fake identification for self-purchasing drinkers was online (47.4%). Of those self-purchasing drinkers with fake identification, males, in particular, were more likely to purchase online (53.7% compared with 36.7% for females, chi-square = 8.8, P = 0.003; df = 1). Other methods included: borrowing identification from an older sibling (21.8% of self-purchasing drinkers); making identification themselves (16.8%) and through family and friends (12.1%). Likelihood of reporting three risky drinking behaviours (binge drinking, public drinking and frequent drinking) was analysed alongside demographic characteristics and ownership of fake identification (Additional File 1). Here, ownership of fake identification amongst drinkers had one of the strongest associations with risky drinking in alcohol consumers: those who owned fake identification were more likely to report binge drinking (AOR = 3.5; 95% CI = 2.8-4.3; P < 0.001; Wald chi-square = 134.2; df = 1), frequent drinking (AOR = 3.0; 95% CI = 2.5-3.7; P < 0.001; Wald chi-square = 117.6; df = 1) and public drinking (AOR = 3.3; 95% CI = 2.5-4.1; P < 0.001; Wald chi-square = 87.9; df = 1) compared with those who did not own fake identification. Those drinkers owning fake identification were also significantly more likely to report experiencing alcohol-related harm, in particular in relation to regretted sex after drinking and entering a car with a drunk driver (Figure 1).

**Table 1:** Estimating the odds of owning of fake identification amongst alcohol consumers from demographic characteristics

		Own at least one form of fake identification						
		
		Univariate Chi square				Logistic regression (n = 6,803; yes = 6.6%)		
		
	df	N	Chi square	%	P ^(a)^	AOR	95% CI	P ^(b)^
Sex								
Female (ref)	1	4,167	53.2	4.6	< 0.001	1.0		< 0.001
Male		3,818		8.6		2.0	1.7-2.5	
Age								
15 (ref)	1	3,872	1.2	6.2	0.270	1.0		0.890
16		4,113		6.8		1.0	0.8-1.2	
Deprivation quintile								
(Least deprived) 1 (ref)	4	1,239	16.1	8.4	0.003	1.0		0.005*
2		1,632		5.1		0.6	0.4-0.8	< 0.001
3		1,385		6.9		0.8	0.6-1.0	0.095
4		1,549		5.4		0.6	0.5-0.8	0.003
(Most deprived) 5		1,883		6.9		0.8	0.6-1.0	0.068
Personal weekly income								
£10 or less (ref)	3	2,495	141.0	3.6	< 0.001	1.0		< 0.001*
£11-20		2,001		5.0		1.4	1.0-1.8	0.045
£21-30		1,005		8.0		2.3	1.7-3.2	< 0.001
More than £30		1,550		12.7		3.7	2.9-4.9	< 0.001

% Univariate percentages are actual rather than expected. df = degrees of freedom. AOR = Adjusted Odds Ratio. Ref = reference category. * P value is for the overall effect of the variable within the logistic regression. Individual P values provided below this compare the individual category with the reference category.

## Discussion

As a cross-sectional survey, this study has several limitations. Sampling techniques excluded young people outside the school education system (such as excludees) and compliance rates from schools were not collected (although response rates to individual questions were over 85% for most questions). Deprivation was assigned to individuals based on ecological classifications rather than an individual's situation. The sample was drawn from a region within the UK known to have high levels of consumption and harm[24]. Thus, it is not recommended that the prevalence estimates are extrapolated to population levels. The analyses relied on self-reported experiences relating to access to alcohol, drinking behaviours and alcohol-related harms. As such, responses could be affected by factors including environmental influences, social desirability and selective recall [30,31]. Anonymity can encourage honesty but behaviours such as underage self-purchase and possession of fake identification are illegal, which may discourage participants from acknowledging their full involvement. Further, memory lapses associated with alcohol use, such as those described by participants of this study, could prevent full recollection both of quantities of alcohol consumed and of subsequent related incidents. The survey specifically examined four alcohol-related harms (entering a car with a drunk driver, memory lapses, regretted sex, and violence), but individuals could have experienced a number of other harms relating to alcohol that were not measured through this survey, such as hospital admission or poor school performance [6,7]. However, the outcomes included did cover a range of different indicators: violence is historically associated with males [32] and provides some information on offending behaviour; regretted sex can be used as a measure of sexual health [33]; memory lapses as a measure of damage to mental health or development [7]; and entering a car with a drunk driver as a measure of risky behaviour. Finally, a further limitation is that the questionnaire did not distinguish between ownership of fake identification for the purpose of accessing alcohol and ownership of fake identification for other purposes (such as to purchase other age-restricted products or to enter a nightclub). Nevertheless, strong relationships were identified between ownership of fake identification, risky drinking behaviours and alcohol-related harms.

Although legislation and interventions have been established to tackle underage drinking and sales in the UK [17,18], notable numbers of underage young people continue to purchase their own alcohol, drink hazardously and experience alcohol-related harm such as violence and regretted sex [8,12,13]. Whilst the most common source for accessing alcohol was through friends/family who were above the legal minimum drinking age, as with a similar study in the United States of America (USA),[34,35] over a quarter (28%) of our drinkers reported that they had purchased alcohol themselves. This is considerably more than in the USA study, where 3% of 14-15 year olds and 9% of 17-18 year olds reported buying alcohol themselves even though they were under the legal minimum age to do so[34]. Even amongst the 18-20 year olds (an age group only just under the minimum purchasing age of 21 years in the USA), only 14% reported self purchase, half the proportion of those who did so in our survey. In our survey, of those who did report self-purchase, 44% reported that they had accessed alcohol without their age ever being checked by alcohol outlets. This is despite increased penalties in the UK for selling alcohol to those aged under 18 years as provided by recent legislation (for example, maximum fines have been raised and licences can be removed) [18] and ongoing work to enforce legislation and improve awareness of the law and the risks to young people [19-21]. Females are less likely to report having been asked for identification than males. This is supported by focus group work performed in the USA, where it was reported that underage females may find it easier to purchase alcohol than males[35]. This may be because females are physically more mature [36] and can alter their apparent age through the use of clothes, hair and make-up to seem older. Because males find it harder to achieve the same effect, they may be more likely to look younger when attempting to self-purchase and so may be more likely to be asked for identification. Of those who purchased their own alcohol, one seventh owned at least one form of fake identification, and the ownership of this was associated with a significantly higher risk both of hazardous drinking patterns and of experiencing a number of alcohol-related harms. Whilst overall the numbers of those with fake identification were small, our findings support American research, which showed that ownership of fake identification has strong associations with heavy drinking [23]. Research to further understand the experiences of those using fake identification could usefully include perceptions of the risk of being caught using fake identification, perceptions of the severity of the outcome of being caught, actual experience of being caught and the outcome on those occasions.

## Conclusions

Young people (aged 15-16 years) who have access to fake identification are at a particularly high risk of reporting hazardous consumption patterns and related harm. Appropriate interventions are urgently needed to tackle consumption amongst this group to prevent underage access to alcohol and to engage with those who attempt to self-purchase. To do so, it is necessary to promote the use of specific age identification formats (such as passports, or in the UK, the PASS scheme), which are harder to copy, as the only methods of identification accepted in alcohol outlets. It may be necessary to seek powers to close websites offering online fake identification, as this was by far the most popular method of accessing fake identification. Other illegal activities are being tackled by closing websites (e.g. illegal football ticket sales [37]). Parents may have a role to play here in a number of ways: educating young people on the risks of excessive underage consumption; monitoring income and spend; and the removal of fake identification. Outside agencies such as retailers, the licensed trade (for example, through door staff), and licensing enforcement agencies may wish to explore the extent to which they can be involved as well. This could include introducing identification scanners to alcohol outlets, which validate the authenticity of identification. In fact, USA research at community festivals suggests that outlets with more restrictive sales policies are associated with a reduced occurrence of underage sales[38]. Enforcement checks that such policies are operational are also important[39]. Finally, policymakers and other stakeholders seeking to reduce alcohol-related harm amongst young people should consider whether interventions could be used at the point at which a young person has attempted to use fake identification to purchase alcohol. This could include campaign material about the harms associated with alcohol consumption but could also include brief interventions and motivational interviewing in the individuals' homes. In the USA, one study has shown that having penalties such as driving licence removal after attempted use of fake identification have been associated with significantly lower proportions of drivers (aged under 21 years) involved in fatal crashes who were over the legal drink drive limit (0.08% blood alcohol concentration)[40]. Such interventions are essential if short- and long-term alcohol-related harms such as hospital admissions [6], criminal offending [8] and alcoholism [11] are to be reduced among young people.

## Competing interests

The authors declare that they have no competing interests.

## Authors' contributions

MM analysed the data and drafted the manuscript. MAB participated in survey design, and MAB and PAC contributed to the data analysis and helped revise the manuscript. LS conducted the survey and edited the manuscript. All authors read and approved the final manuscript.

## Supplementary Material

Additional file 1 **Estimating the odds of risky drinking amongst alcohol consumers from demographics and ownership of fake identification**. A table in a Word document. Click here for file
