# Supplementary material for: Use of fake identification to purchase alcohol amongst 15-16 year olds: a cross-sectional survey examining alcohol access, consumption and harm
Source: Subst Abuse Treat Prev Policy. 2010 Jun 22;5:12. doi: 10.1186/1747-597X-5-12 (PMC2898792; doi:10.1186/1747-597X-5-12)
Supplement: Additional file 1 — Estimating the odds of risky drinking amongst alcohol consumers from demographics and ownership of fake identification. A table in a Word document. [file 1747-597X-5-12-S1.DOC]

**Additional File 1 - Estimating the odds of risky drinking amongst alcohol consumers from demographics and ownership of fake identification**

|  | df | Binge drinking | | | | Frequent drinking | | | | Public drinking | | | |
| --- | --- | --- | --- | --- | --- | --- | --- | --- | --- | --- | --- | --- | --- |
|  |  | Univariate Chi square | | Logistic regression  (n=6,744; yes=37.1%) | | Univariate Chi square | | Logistic regression  (n=6,800; yes=29.5%) | | Univariate Chi square | | Logistic regression  (n=6,803; yes=57.1%) | |
|  |  | % | P | AOR (95% CI) | P | % | P | AOR (95% CI) | P | % | P | AOR (95% CI) | P |
| Sex |  |  |  |  |  |  |  |  |  |  |  |  |  |
| Female (ref) | 1 | 34.3 | <0.001 | 1.0 | 0.006 | 25.5 | <0.001 | 1.0 | <0.001 | 57.1 | <0.001 | 1.0 | <0.001 |
| Male |  | 38.4 |  | 1.2 (1.0-1.3) |  | 32.1 |  | 1.3 (1.2-1.4) |  | 53.2 |  | 0.8 (0.7-0.9) |  |
| Age |  |  |  |  |  |  |  |  |  |  |  |  |  |
| 15 (ref) | 1 | 32.9 | <0.001 | 1.0 | <0.001 | 26.7 | <0.001 | 1.0 | 0.029 | 52.4 | <0.001 | 1.0 (1.0-1.0) | 0.007 |
| 16 |  | 39.5 |  | 1.2 (1.1-1.4) |  | 30.5 |  | 1.1 (1.0-1.6) |  | 57.9 |  | 1.1 (1.0-1.3) |  |
| Deprivation quintile |  |  |  |  |  |  |  |  |  |  |  |  |  |
| (Least deprived) 1 (ref) | 4 | 31.1 | <0.001 | 1.0 | <0.001* | 28.5 | 0.037 | 1.0 | 0.005* | 53.3 | 0.002 | 1.0 | 0.002* |
| 2 |  | 34.5 |  | 1.2 (1.0-1.4) | 0.027 | 26.9 |  | 1.1 (0.9-1.3) | 0.312 | 52.6 |  | 1.0 (0.9-1.2) | 0.976 |
| 3 |  | 36.3 |  | 1.3 (1.1-1.5) | 0.011 | 29.7 |  | 1.3 (1.1-1.6) | 0.004 | 59.1 |  | 1.3 (1.1-1.6) | 0.001 |
| 4 |  | 37.5 |  | 1.4 (1.2-1.6) | <0.001 | 29.8 |  | 1.3 (1.1-1.6) | 0.002 | 54.5 |  | 1.1 (1.0-1.3) | 0.116 |
| (Most deprived) 5 |  | 39.1 |  | 1.4 (1.2-1.7) | <0.001 | 29.6 |  | 1.3 (1.1-1.5) | 0.008 | 55.2 |  | 1.2 (1.0-1.4) | 0.029 |
| Personal weekly income |  |  |  |  |  |  |  |  |  |  |  |  |  |
| £10 or less (ref) | 3 | 27.4 | <0.001 | 1.0 | <0.001* | 21.4 | <0.001 | 1.0 | <0.001* | 48.8 | <0.001 | 1.0 | <0.001* |
| £11-20 |  | 39.6 |  | 1.7 (1.5-2.0) | <0.001 | 30.6 |  | 1.6 (1.4-1.8) | <0.001 | 57.5 |  | 1.4 (1.3-1.6) | <0.001 |
| £21-30 |  | 39.3 |  | 1.7 (1.4-1.9) | <0.001 | 31.6 |  | 1.7 (1.4-2.0) | <0.001 | 57.4 |  | 1.4 (1.2-1.6) | <0.001 |
| More than £30 |  | 48.4 |  | 2.3 (2.0-2.6) | <0.001 | 40.1 |  | 2.2 (1.9-5.6) | <0.001 | 68.0 |  | 2.1 (1.8-2.4) | <0.001 |
| Ownership of fake identification |  |  |  |  |  |  |  |  |  |  |  |  |  |
| No (ref) | 1 | 34.3 | <0.001 | 1.0 | <0.001 | 26.9 | <0.001 | 1.0 | <0.001 | 54.0 | <0.001 | 1.0 | <0.001 |
| Yes |  | 67.6 |  | 3.5 (2.8-4.3) |  | 57.6 |  | 3.0 (2.5-3.7) |  | 81.3 |  | 3.3 (2.5-4.1) |  |

% Univariate percentages are actual rather than expected. df = degrees of freedom. AOR = Adjusted Odds Ratio. Ref = reference category. For drinking behaviours, binge drinking is defined as consuming five or more alcoholic drinks on one occasion, frequent drinking is defined as consuming alcohol at least twice per week, and drinking in public places is defined as consuming alcohol in streets, around shops and in parks. *For logistic regression P values, those alongside the referent category indicate overall significance for the variable, while below compare the individual category with the reference category.
